# Assessment of Externally Prestressed Beams with FRP Rebars Considering Bond–Slip Effects †

**DOI:** 10.3390/ma18040787

**Published:** 2025-02-11

**Authors:** Zhangxiang Li, Bo Chen, Xueliang Wang, Tiejiong Lou

**Affiliations:** 1 Sanya Science and Education Innovation Park, Wuhan University of Technology, Sanya 572025, China; 306634@whut.edu.cn (Z.L.); whutchenbo@gmail.com (B.C.); wxllhb@163.com (X.W.); 2 School of Civil Engineering and Architecture, Wuhan University of Technology, Wuhan 430070, China; 3 CEMMPRE, ARISE, Faculty of Sciences and Technology, University of Coimbra, 3030-788 Coimbra, Portugal

**Keywords:** fiber-reinforced polymer, bond–slip, externally prestressed concrete beams, finite element model, tendon stress

## Abstract

This paper presents detailed numerical modeling of externally prestressed concrete (EPC) beams with fiber-reinforced polymer (FRP) rebars. Particular attention is paid to the bond–slip interactions between FRP rebars and concrete. A refined 3D finite element model (FEM) incorporating a script describing the bond–slip of FRP rebars and concrete is developed in ABAQUS. The model effectiveness, rooted in the interface behavior between FRP rebars and concrete, is comprehensively assessed using experimental data. A comprehensive investigation has been conducted using FEM on the mechanical behavior of carbon fiber-reinforced polymer (CFRP) tendon–EPC beams with FRP rebars. Due to the bond–slip effect, FRP rebars in EPC beams exhibit a distinct phenomenon of stress degradation. This suggests that the traditional method based on plane cross-sectional assumptions is no longer suitable for the engineering design of EPC beams with FRP rebars. Moreover, the study assesses several models including typical design codes for their accuracy in predicting the elevation of ultimate stress in external tendons. It is demonstrated that some of the design codes are overly conservative when estimating the tendon stress in EPC beams with FRP rebars.

## 1. Introduction

External prestressing offers many benefits, such as enhanced structural capacity [1], reduced cracking with improved shear strength [2], easy replacement of external tendons, and minimal friction loss [3], among others. Performance degradation caused by the corrosion of traditional steel reinforcement is still an issue that cannot be ignored [4]. Replacing steel with fiber-reinforced polymer (FRP) materials in engineering structures is an effective measure to solve the corrosion problem [5,6]. FRP is defined by its low density, high tensile strength, and limited fire resistance [7]. FRP composites exhibit linear elastic behavior without a defined yield point [3,8]. These materials have demonstrated effectiveness as either bonded rebars in concrete or external tendons for bridge structures [9]. In civil engineering, various FRP rebars, including basalt (BFRP) [10], carbon (CFRP) [11,12], and glass (GFRP) [13], are applied. CFRP, in particular, provides excellent resistance to creep rupture, making it well-suited for prestressing applications [14,15].

Researchers have broadly examined the application of CFRP tendons in externally prestressed concrete (EPC) beams. Grace and Abdel [16] evaluated four CFRP tendon–EPC beam models under static and cyclic loading (7 million cycles), showing that CFRP tendons enhanced the structural ductility and induced subsurface elastic deformation, which might lead to compression failure. Yu et al. [17] proposed a 10° limit for the bending angle of external CFRP tendons and investigated the relationship between reinforcement ratio, load capacity, and beam flexibility. They also proposed a simplified equation for calculating flexural capacity [17]. Ghallab and Beeby [18] and Ghallab [19] explored the impact of tendon type, eccentricity-to-depth ratio, and deviator placement on external tendon stress through experimental and theoretical analysis. The finite element model (FEM) has been successfully used in the structural design and analysis [20]. By applying FEM, Duan et al. [21] studied the static performance of two EPC beams under symmetric and unsymmetric loads and proposed a simplified formula for estimating the ultimate moment modulation coefficient. Tran et al. [22] proposed a FEM to simulate segmented precast beams with external CFRP cables, addressing the role of joint openings and deviator locations in second-order effects. Based on FEM, Pang et al. [23] and Lou et al. [24] assessed CFRP tendon–EPC beams with FRP rebars, evaluated several existing design codes, and introduced predictive models for ultimate tendon stress. However, their models assumed a fully bonded interaction between rebars and concrete, leading to an overestimation of the stress in rebars and the flexural strength of the beams.

The behavior of FRP rebars in concrete depends upon attributes such as the geometry and surface texture of the rebars, concrete strength, rebar diameter, embedded length, and environmental conditions [25]. The bonding characteristic between embedded FRP rebars and concrete has been extensively studied [26,27]. Xue et al. [28] concluded from pull-out tests on 84 sets of sand-coated deformed GFRP bars that splitting failure occurs when the embedded depth exceeds five times the bar diameter. Their findings indicated that bond strength rose with enhanced concrete strength but diminished as the rebar diameter grew. Fahmy et al. [29] reported that BFRP rebars with damaged surfaces performed better in bond tests compared to other BFRP and steel rebars. Lin and Zhang [30] developed a composite beam model and performed finite element analysis on CFRP and GFRP-reinforced concrete beams using bond stress/slip models. Considerable studies have focused on the typical bond stress/slip relationship between FRP rebars and concrete [31,32,33].

To contribute to a deep insight into the bond–slip effects of FRP rebars in EPC beams, this study introduces a refined 3D FEM for EPC beams with FRP rebars, taking into account their interaction with concrete. The bond–slip model and FEM are validated by available test results. A comprehensive numerical analysis of simple CFRP tendon-EPC beams is conducted. Key variables investigated include the type of bonded rebars (CFRP, GFRP, and steel), GFRP elastic modulus, concrete grade, and tensile reinforcement ratio. Additionally, five existing models in predicting the ultimate external tendon stress increment are evaluated.

## 2. FEM and Verification

### 2.1. Material Property and Bond Stress–Slip

**Figure 1:**
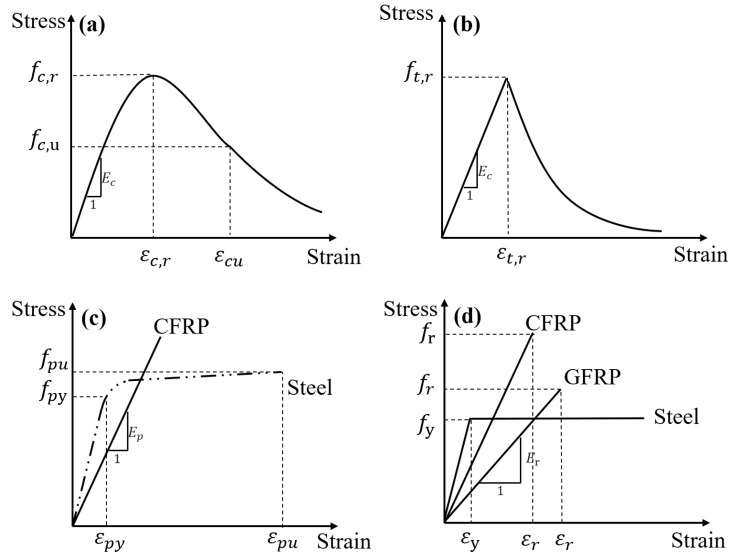
Stress–strain responses for the materials employed. (**a**) concrete under compression; (**b**) concrete under tension; (**c**) prestressing tendons; (**d**) bonded rebars.

Figure 1 demonstrates the stress–strain responses of materials used for theoretical purposes. Concrete at compression and tension is modeled according to GB 50010-2010 [34] (see Figure 1a,b). CFRP tendons behave elastically until rupture, whereas steel tendons follow a constitutive law represented by the Menegotto and Pinto [35] approach (Figure 1c). FRP rebars exhibit elastic behavior up to rupture, whereas steel rebars follow an elastic–perfectly plastic model (Figure 1d). In Figure 1, *ε_c,r_* = concrete compressive strain at peak stress *f_c,r_* (concrete cylinder compressive strength); *E_c_* = concrete elastic modulus; *ε_c,u_* = ultimate concrete compressive strain at stress *f_c,u_* = 0.5 *f_c,r_*; *ε_t,r_* = concrete cracking strain at tensile strength *f_t,r_*; *ε_pu_* = steel tendon ultimate tensile strain at tensile strength *f_pu_*; *ε_py_* = steel tendon yield strain at yield strength *f_py_*, normally taken as 0.94 *f_pu_* in model; *E_p_* and *E_r_* = tendon and rebar elastic modulus, respectively; *f_r_* = FRP rebar rupture strength; *f_y_* and *ε_y_* = yield strength and strain of steel rebars, respectively.

**Figure 2:**
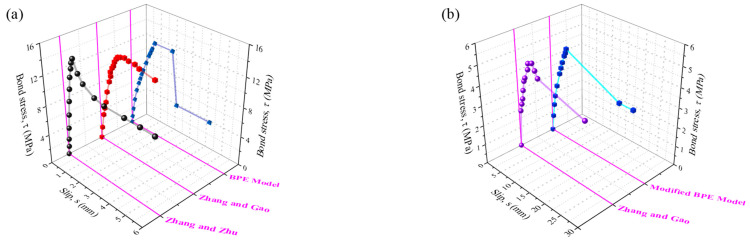
Bond stress–slip graphs of FRP rebars and concrete. (**a**) ribbed FRP; (**b**) plain FRP [31,32,36,37].

Figure 2 presents the bond stress–slip curves for ribbed and plain FRP rebars derived from pull-out tests [36,37] and typical models [31,32]. The bond strength *τ*, namely the mean bond stress over the embedded length, is determined by dividing the pull-out load by the surface area of embedded FRP bars, as given by Equation (1):

(1) $$\tau =P/(\pi d_{f}l_{fb})$$

where *P* denotes the pull-out load applied to the specimen; *d_f_* and *l_fb_* are the diameter and embedded length of FRP rebars in concrete, respectively.

Zhang and Zhu [36] conducted central pull-out tests on 84 FRP-reinforced concrete samples, examining the differential stress state of the specimens to derive the bond stress–slip relationship for typical FRP rebars and concrete. In a subsequent study, Zhang and Gao [37] tested 17 additional specimens, with bond failure in 7 groups and FRP bar fracture in the rest. The bond–slip profiles of ribbed and plain FRP rebars were obtained. As shown in Figure 2, the experimental data closely align with the predictions made by the BPE (Bertero–Popov–Eligehausen) [31] and Modified BPE [32] models, despite the absence of the bond–slip curves in the residual stage.

### 2.2. Bond Stress–Slip Model Validation

Three reinforced concrete beams with FRP and steel rebars, reported by Xu et al. [38], were analyzed. The beams, labeled BL1-2, TL1-2, and BL2-1, were categorized based on the size and surface treatment of the tensile FRP rebars (ribbed and plain) and tested using a four-point loading scheme until structural failure. The diameter, elastic modulus, and rupture strength were 9.5 mm, 40 Gpa, and 606 MPa, respectively, for plain GFRP (P-GFRP) rebars in BL1-2; 9.5 mm, 136 GPa and 1779 MPa, respectively, for ribbed CFRP (R-CFRP) rebars in TL1-2; and 9.5 mm, 72 GPa, and 993 MPa, respectively, for ribbed GFRP rebars in BL2-1. Other material parameters are listed in Table 1. More details of the specimens can be seen in [38].

**Figure 3:**
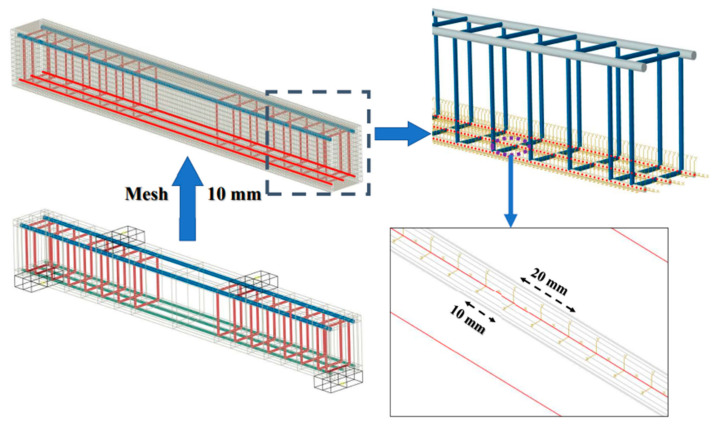
Assignment in mesh attribute and connector of specimens.

Numerical verification of the load-deflection behavior of the specimens is carried out by proposing ABAQUS FEM [39]. Concrete is modeled with solid hexahedral elements (C3D8R), rebars with 2-node linear 3D truss elements (T3D2), and the pad with 4-node bilinear 3D rigid bodies (R3D4). The contact area between the concrete and FRP rebars is discretized into 10 mm mesh elements, with the mesh size selected based on convergence. A custom-designed program is employed to create connectors between the contact nodes of the two components, as illustrated in Figure 3. By creating a connector section and applying the bond vs. slip curves between FRP rebars and concrete, the relative sliding under loading could be accurately simulated. The bond force *F_b_* is determined by Equation (2) as follows:

(2) $$F_{b}=\pi D\tau d_{x}$$

where *D* is the FRP rebar diameters; *τ* and *d_x_* are the bond stress and length of mesh elements, respectively.

**Figure 4:**
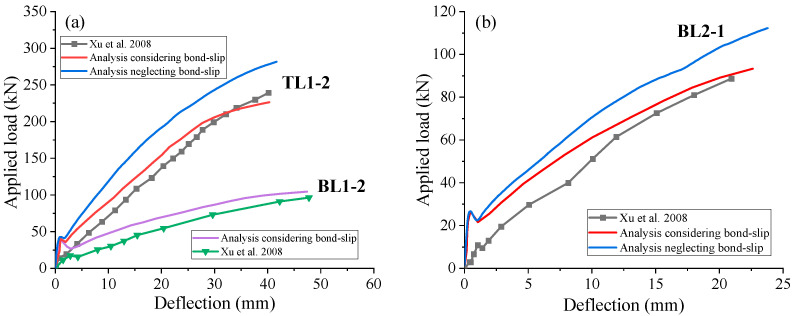
Comparison with test data for bond–slip model validation. (**a**) C1; (**b**) C2 [38].

A comparison of the load-deflection curves obtained from FEM and tests is illustrated in Figure 4. Table 2 compares the experimental and numerical results regarding the ultimate load and deflection for the specimens. It is evident that the bond–slip effect of FRP rebars plays a critical role in the FEM results. The FEM neglecting bond–slip effect results in 24.4% and 20.3% higher bearing capacity than the FEM considering bond–slip effect for TL1-2 and BL2-1, respectively. The FEM results indicate that the full strength of the FRP rebars is not utilized in BL1-2 due to the use of plain FRP rebars, which exhibit a lower elastic modulus and an insufficient bonding with concrete. This leads to a significant increase in beam deflection and sliding failure of the FRP rebar. Although there are some differences, the FEM predictions (considering bond–slip effect) closely match the experimental data across the entire loading range.

Note that the bond–slip behavior is modeled using a predefined relationship based on idealized experimental data. This approach assumes uniform material behavior and neglects potential variations due to environmental conditions or microstructural differences. Consequently, such assumptions may lead to deviations in predicting localized stress distributions, especially under complex loading conditions.

### 2.3. Verification with Experimental Results of EPC Beams

**Figure 5:**
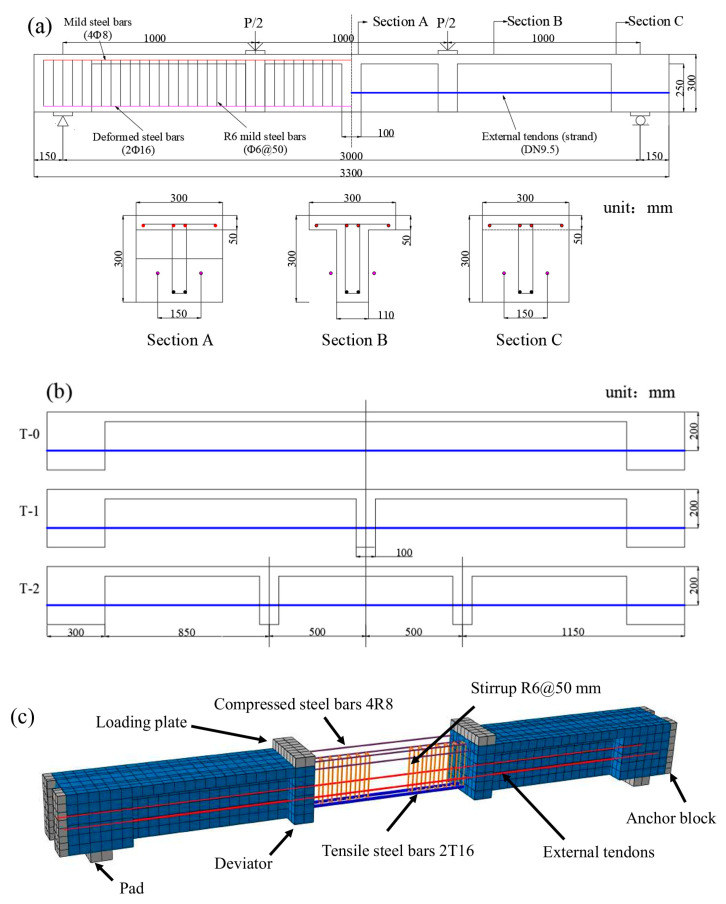
Simple EPC beams. (**a**) dimensions and rebar details; (**b**) external tendon configuration; (**c**) FEM mesh of T-2.

Tan and Ng [40] carried out several experiments to investigate the behavior of EPC beams. The deviator arrangement, tendon eccentricity, and effective prestress were the primary testing factors. Li et al. [41] developed a refined three-dimensional FEM incorporating both material and geometric nonlinearities of the structure. This model was validated by analyzing the deflection and tendon stress increments of three specimens (T-0, T-1, and T-2) under varying loads and comparing the results with experimental data [41]. The structure and reinforcement details of the specimens are shown in Figure 5a,b, and the material properties are detailed in Table 3.

The FEM mesh (Beam T-2) is shown in Figure 5c. Concrete elements are modeled using solid hexahedral elements (C3D8R), while steel rebars and tendons are represented by 2-node linear 3D truss elements (T3D2). The plate and pad are modeled using 4-node bilinear 3D rigid bodies (R3D4). The beam–pad interface is modeled as a tie contact, preventing slippage. The effective prestress could be predicted by applying a temperature differential between the initial and target temperature steps [42]. The effective prestress, *σ_pe_*, is conventionally calculated using Equation (3) as follows:

(3) $$\sigma_{pe}=\lambda \Delta T_{t}E_{p}A_{p}$$

where *λ* is the thermal expansion factor; $\Delta T_{t}$ is the temperature difference between the initial and next step; *E_p_* and *A_p_* are the Young’s modulus and area of external tendons, respectively.

**Figure 6:**
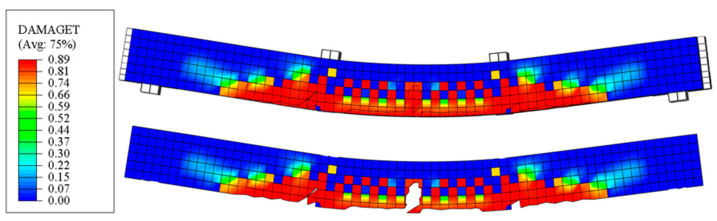
Damage nephograms of T-2.

Figure 6 shows the damage distribution of Beam T-2, illustrating the formation and progression of concrete micro-defects during service. The upper figure is the middle longitudinal section of T-2, while the lower figure is the edge longitudinal section only showing the concrete. Concrete failure in ABAQUS is represented by a damage parameter, which ranges from 0 (unloaded) to 1 (fully degraded). The damage nephograms indicate that concrete failure initiates at midspan after significant tensile rebar yielding. This aligns with the experimentally observed failure mechanism of Beam T-2.

**Figure 7:**
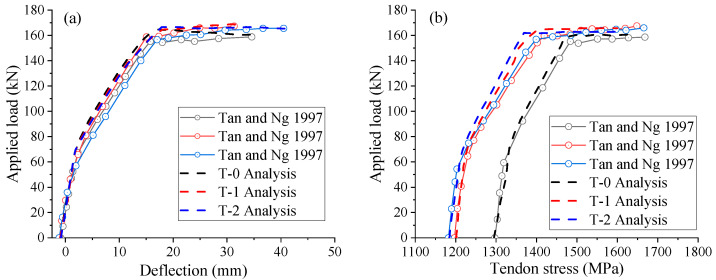
Validation against tests. (**a**) load vs. midspan deflection; (**b**) load vs. tendon stress [40].

Figure 7 compares the load-deflection and load-tendon stress curves of T-beams generated by the proposed FEM with the experimental data. After concrete cracking, the FEM-simulated load-deflection curves show a bit stiffer behavior than the experimental results but follow a similar pattern. While the FEM results show an underestimation of the ultimate tendon stress in all specimens, the numerical outcomes are consistent with the experimental data across the entire loading range. Table 4 compares the experimental and numerical results regarding the ultimate deflection and tendon stress, confirming the reliability of the FEM in modeling EPC beams.

## 3. FEM Assessment

**Figure 8:**
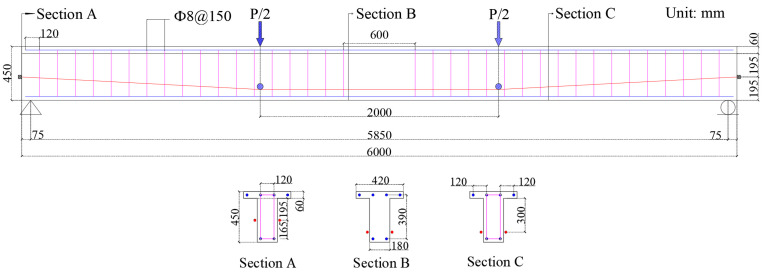
Externally prestressed T-beams for numerical assessment.

This study examines simple CFRP tendon–EPC T-beams under four-point loading, as illustrated in Figure 8. A simply supported condition is considered using nonlinear static analyses with the full Newton method. Tendon depths are 255 mm at anchorage points and 360 mm at deviator points. The material parameters are chosen in accordance with the current design code [9]. CFRP tendons have a tensile strength of 1927 MPa, Young’s modulus of 160 GPa, a cross-sectional area of 226.08 mm^2^, and an initial prestress of 1060 MPa (namely 55% of tensile strength). Stirrups are made of 8 mm diameter CFRP bars with Young’s modulus of 147 MPa. The area of four compressive rebars is 314 mm^2^. The area of two tensile rebars, *A_r_*, ranges from 402 to 1608 mm^2^. The ratio of tensile rebars (*ρ_r_*), which is no less than 0.22% in T-beam, can be precisely determined by Equation (4) as follows:

(4) $$\rho_{r}=\frac{A_{r}}{d_{r}b_{f}^{\prime }-(b_{f}^{\prime }-b)(d_{r}-h_{f}^{\prime })}$$

where *A_r_* is the tensile rebar area; $b_{f}^{\prime }$ and *b* are the flange width and web thickness, respectively; $h_{f}^{\prime }$ and *d_r_* are the flange thickness and the depth of tensile rebars, respectively.

The anchorage length of FRP bars, typically determined through pull-out tests of reinforcement and concrete, plays a crucial role in the bond performance. The embedded length of FRP rebars should be at least four times their diameter [36]. The effects of stirrup spacing and bending angle on shear behavior are significant. When the shear span-to-depth ratio exceeds 2.5, flexural behavior becomes the primary concern, while shear effects are negligible [34]. Given that the shear span-to-depth ratio in this EPC beam model is 4.4, the study primarily focuses on flexure-related parameters.

**Figure 9:**
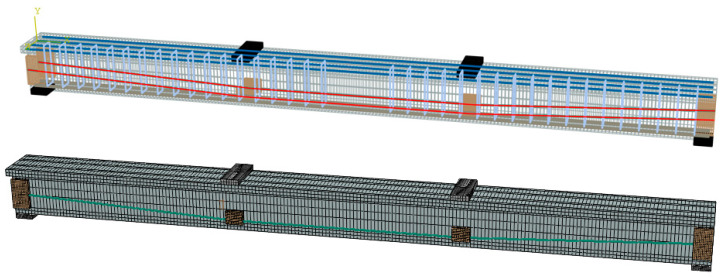
FEM mesh of EPC beams.

**Figure 10:**
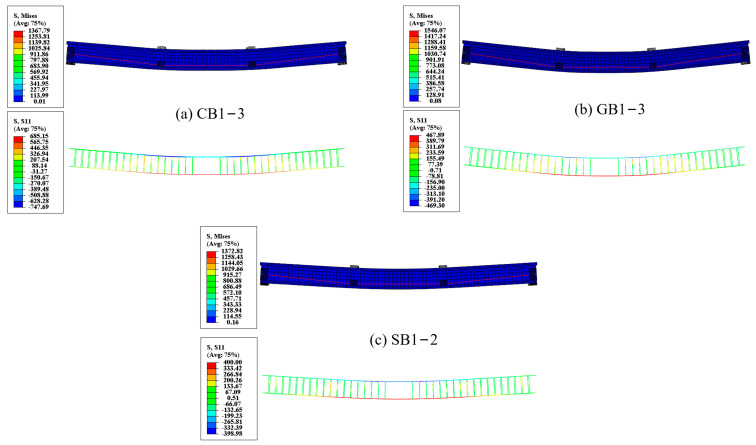
Nephograms of structure and bond rebars for typical beams.

Three groups of 21 EPC beams are established, with the variables including the rebar type (CFRP, GFRP, and steel), FRP elastic modulus (40–70 GPa), concrete grade (C30–C50), and reinforcement ratio of tensile rebars (0.50–1.98%). The design parameters for all EPC beams are summarized in Table 5, with reference beams designated as CB1-3, GB1-3, and SB1-2. Based on the characteristics of these reference beams, additional factors are examined through comparative studies. The mesh size (30 mm) for the FEM of EPC beams is illustrated in Figure 9, and the Mises stress and bond rebar stress distribution for typical beams are shown in Figure 10. Typical results regarding the ultimate behavior are presented in Table 6.

### 3.1. Effect of Rebar Type

**Figure 11:**
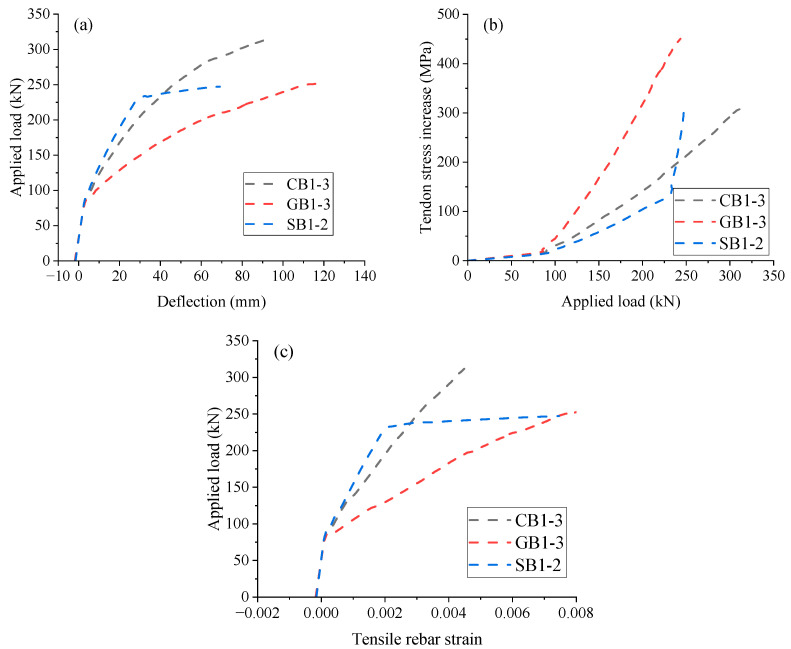
Effect of rebar type. (**a**) midspan deflection vs. load; (**b**) load vs. tendon stress increase; (**c**) tensile rebar strain vs. load.

Figure 11a presents the load vs. midspan deflection development of EPC beams with different rebar types (CFRP, GFRP, and steel). The curve of Beam SB1-2 (steel rebars) can be segmented into three phases, identified by critical points of concrete cracking and steel yielding. Notably, a marked reduction in flexural stiffness is observed upon yielding. EPC beams with FRP rebars exhibit a linear-to-curved response, with the transition caused by concrete cracking. This curved behavior can be attributed to the bond–slip interaction between FRP rebars and concrete. As demonstrated in Figure 11a and Table 6, the ultimate load of SB1-2 (steel rebars) is substantially lower than that of CB1-3 (CFRP rebars) but nearly comparable to that of GB1-3 (GFRP rebars). Meanwhile, Beam GB1-3 exhibits substantially greater ultimate deflection than Beam SB1-2, suggesting that GFRP rebars are ideal substitutes for steel rebars in EPC beams to increase the deformation capacity without compromising the ultimate load-carrying capacity.

Figure 11b,c depicts the load vs. tendon stress increase and load vs. tensile rebar strain for EPC beams with different rebar types, respectively. As FRP rebars do not yield, Beam CB1-3 and GB1-3 exhibit a bilinear response, with the turning point corresponding to concrete cracking, as shown in Figure 11b. In contrast, Beam SB1-2 displays a trilinear response characterized by cracking and yielding. The type of rebar has a minimal influence on tendon stress development within the elastic range, as it is primarily controlled by concrete. After cracking, GB1-3 experiences a smaller rebar stress increase due to the lower elastic modulus of GFRP rebars, necessitating a larger external tendon stress increase to maintain equilibrium compared to CB1-3 and SB1-2. The tensile rebar strain behavior for the beams resembles each other in the early stage but differs thereafter. Steel rebars, before yielding, exhibit a slower increase in tensile strain, and, after yielding, the development turns out to be much quicker when compared to FRP rebars. The ultimate tensile strain in steel rebars is substantially greater than that in CFRP rebars, while it appears to be comparable to that in GFRP rebars, as illustrated in Figure 11c and Table 6.

**Figure 12:**
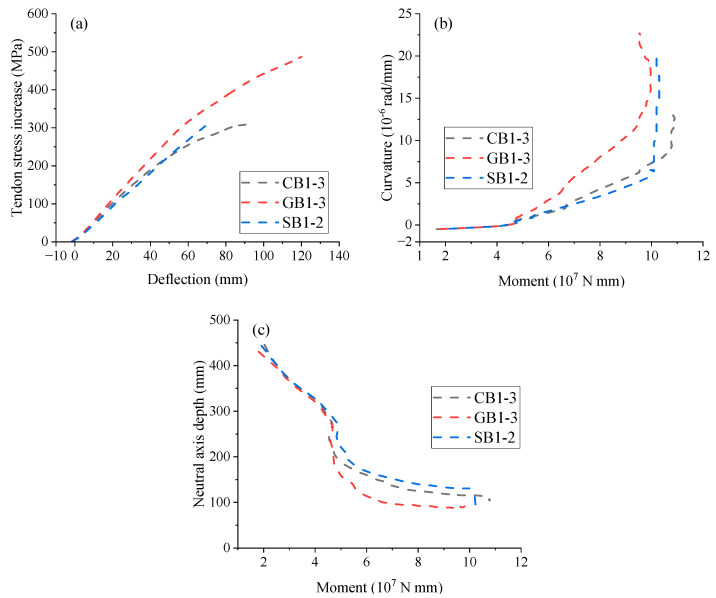
Effect of rebar type. (**a**) deflection vs. tendon stress increment; (**b**) moment vs. curvature; (**c**) moment vs. neutral axis depth.

Figure 12a shows the development of tendon stress increment vs. midspan deflection for EPC beams with different rebar types. The bond–slip effect between FRP rebars and concrete leads to nonlinear tendon stress development in CB1-3 and GB1-3. In contrast, SB1-2 shows nearly linear behavior because of the perfect bond behavior of steel rebars assumed in the FEM. Figure 12b shows the moment vs. midspan curvature curves for EPC beams with different rebar types. Due to bond–slip effects between FRP rebars and concrete, the post-cracking curvature of beams with FRP rebars deviates from linearity, following an arc-like growth before decreasing at failure. The earlier onset of this behavior in GB1-3 suggests earlier stiffness degradation, as the lower elastic modulus of GFRP causes more reduction in structural stiffness after concrete cracking. At ultimate, GB1-3 displays the largest curvature, which is 1.69 and 1.11 times that of CB1-3 and SB1-2, respectively, indicating favorable deformation capacity. Figure 12c illustrates the moment-neutral axis depth behavior at midspan for EPC beams with different rebar types. In beams with FRP rebars (CB1-3 and GB1-3), the neutral axis initially experiences a rapid shift as the moment increases, followed by a gradual deceleration. A similar trend is observed in SB1-2 until yielding, after which the neutral axis movement accelerates.

### 3.2. Effect of GFRP Modulus of Elasticity

**Figure 13:**
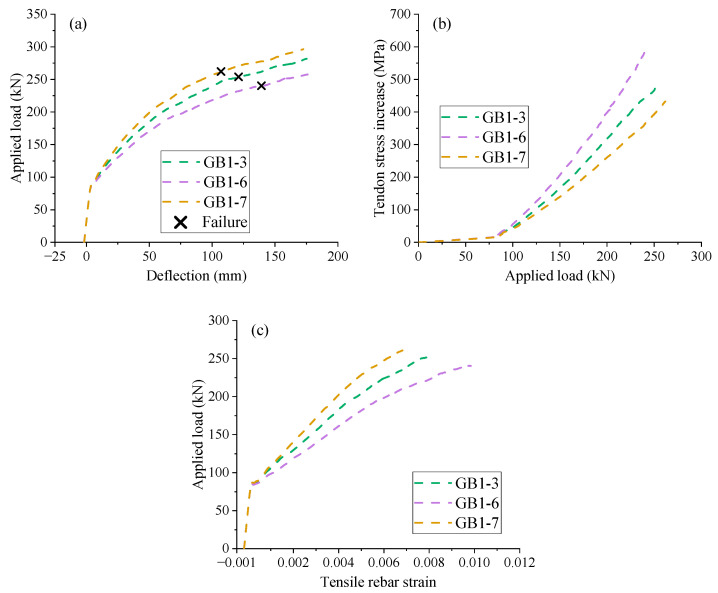
Effect of GFRP rebar elastic modulus. (**a**) midspan deflection vs. load; (**b**) load vs. tendon stress increase; (**c**) tensile rebar strain vs. load.

Three EPC beams with GFRP rebars having different *E_r_* (elastic modulus of rebars) values are used, i.e., GB1-3 (*E_r_* = 55 GPa), GB1-6 (*E_r_* = 40 GPa), and GB1-7 (*E_r_* = 70 GPa). Figure 13a shows that prior to cracking, the three beams share the same load-deflection behavior due to the negligible role of rebars. After cracking, the rebars play a more and more important role, and therefore, the structural stiffness of the beams is strongly dependent on the GFRP elastic modulus, namely, the largest structural stiffness occurring in GB1-7 and the smallest one in GB1-6. At failure, which is marked in the graphs of Figure 13a, the ultimate load of GB1-7 is 1.04 times that of GB1-3 and 1.09 times that of GB1-6, while the ultimate deflection of GB1-7 is 89.75% and 76.51% of that of GB1-3 and GB1-6, respectively. Figure 13b shows that for the same post-cracking load levels, GB1-7 (*E_r_* = 70 GPa) demonstrates the lowest external tendon stress while GB1-6 (*E_r_* = 40 GPa) exhibits the highest one. An explanation is that a higher elastic modulus in the GFRP rebars leads to a higher rebar stress, although it causes a lower rebar strain, as shown in Figure 13c, consequently resulting in a smaller tendon stress according to the section force equilibrium.

### 3.3. Effect of Concrete Grade

**Figure 14:**
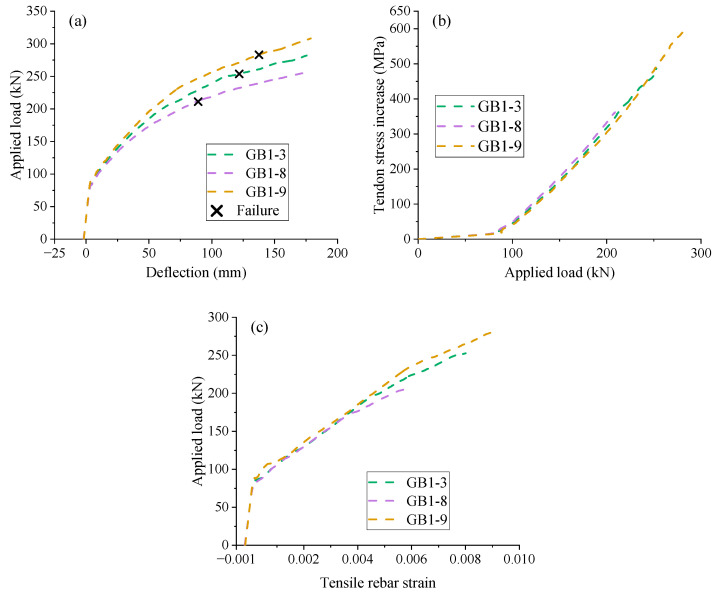
Effect of concrete grade. (**a**) midspan deflection vs. load; (**b**) load vs. tendon stress increase; (**c**) tensile rebar strain vs. load.

To examine the influence of concrete grade, three EPC beams with GFRP rebars and different concrete grades are selected, namely, GB1-3 (C40), GB1-8 (C30), and GB1-9 (C50). Figure 14a presents the load vs. midspan deflection development of the beams. A higher concrete grade results in stiffer post-cracking behavior, attributed to not only the higher concrete elastic modulus but also the better exploitation of reinforcement materials. As illustrated in Figure 14a and Table 6, the ultimate load and deflection of GB1-9 (C50) are 37.17% and 59.91%, respectively, higher than those of GB1-8 (C30). However, the effect of concrete grade varies depending on the type of rebars used. For example, if steel rebars are used, the influence of concrete grade on the ultimate load of the beams may not be so important because the steel rebars have usually yielded prior to the ultimate limit state. Figure 14b,c depicts the load vs. tendon stress increase and load vs. tensile rebar strain curves of the beams, respectively. An increase in concrete grade from C30 to C50 results in approximately a 25% rise in tendon stress increment. Importantly, the slopes of the load vs. tendon stress increase curves for the three beams remain almost consistent, equal to 2.76 MPa/kN, as shown in Figure 14b. In addition, increasing the concrete grade significantly increases the ultimate tensile strain of FRP rebars, as illustrated in Figure 14c and Table 6. For example, the tensile rebar strain of GB1-3 (C40) and GB1-9 (C50) is 33.33% and 51.67%, respectively, greater than that of GB1-8 (C30).

**Figure 15:**
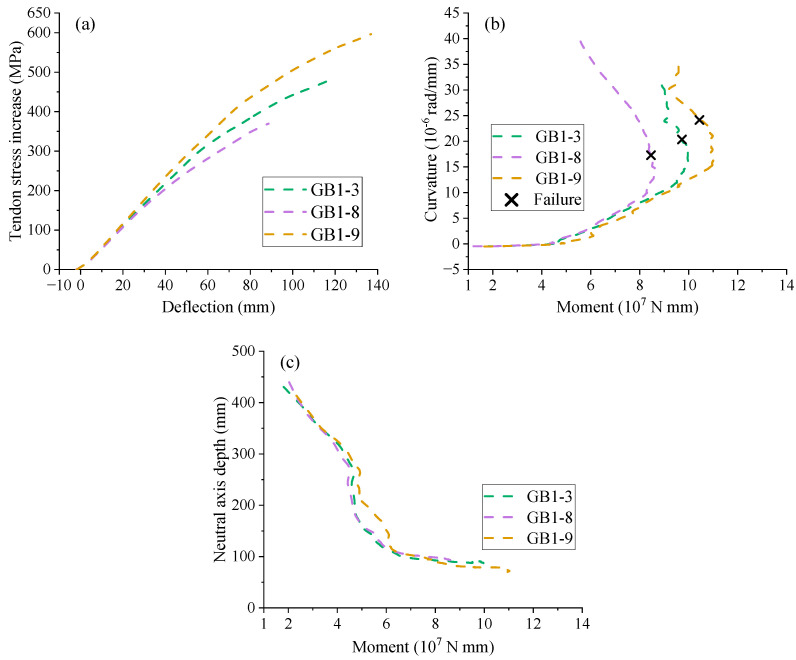
Effect of concrete grade. (**a**) deflection vs. tendon stress increment; (**b**) moment vs. curvature; (**c**) moment vs. neutral axis depth.

Figure 15a illustrates the tendon stress development against the midspan deflection of the beams. The curves in the figure exhibit an approximately linear relationship; however, due to the slip between the GFRP rebars and concrete, a gradual decrease in the slope occurs in the later stage. The moment-curvature behavior of Figure 15b shows that increasing the concrete grade enhances the bending stiffness after cracking. In addition, a higher concrete grade corresponds to greater ultimate curvature and bending moment. Figure 15c illustrates that the concrete grade does not have a significant impact on the neutral axis evolution during loading. However, at ultimate, a high concrete grade corresponds to a smaller neutral depth, as illustrated in Figure 15c and Table 6.

### 3.4. Effect of Reinforcement Ratio

**Figure 16:**
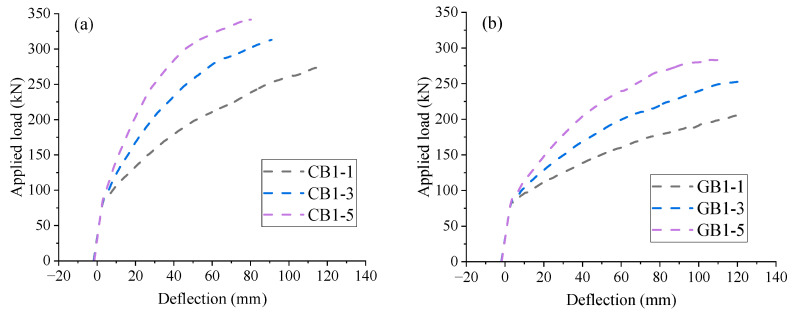
Effect of reinforcement ratio on the load-deflection behavior. (**a**) EPC beams with CFRP rebars; (**b**) EPC beams with GFRP rebars.

The load-deflection behavior of EPC beams with different FRP reinforcement ratios (*ρ_r_* = 0.50–1.98%) is shown in Figure 16. As expected, the reinforcement ratio does not impact the elastic behavior until cracking. Thereafter, the higher the reinforcement ratio is, the higher the structural stiffness is. As mentioned previously, nonlinear post-cracking load-deflection behavior arises from the relative slip between FRP rebars and concrete. However, the degree of nonlinearity for CB1-5 or GB1-5 (*ρ_r_* = 1.98%) appears to be more severe than that for CB1-1 or GB1-1 (*ρ_r_* = 0.50%). The lower bond strength observed in CB1-5 or GB1-5 is due to the use of CFRP or GFRP rebars with a larger diameter.

**Figure 17:**
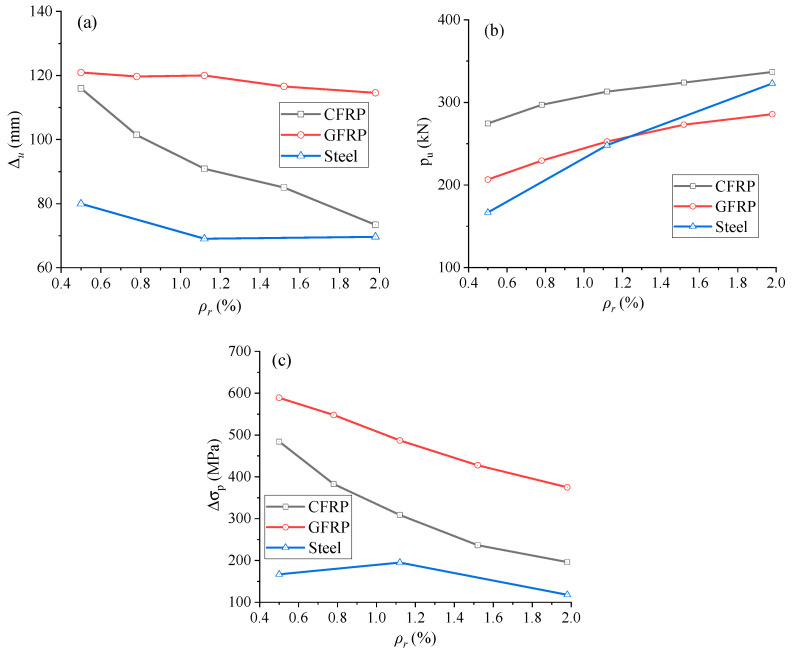
Ultimate behavior with varying reinforcement ratio. (**a**) ultimate deflection; (**b**) ultimate load; (**c**) ultimate tendon stress.

Figure 17a illustrates the relationship between ultimate midspan deflection (Δ*_u_*) and the tensile reinforcement ratio (*ρ_r_*). At *ρ_r_* = 0.5%, EPC beams with CFRP rebars show slightly lower ultimate deflection compared to those with GFRP rebars. By increasing *ρ_r_*, the ultimate deflection of EPC beams with FRP rebars generally decreases, with the reduction being more pronounced in beams with CFRP rebars than those with GFRP rebars. Beams with GFRP rebars exhibit significantly higher ultimate deflection (51.24–73.73%) than those with steel bars, and the deflection difference between EPC beams with CFRP and GFRP rebars widens with increasing *ρ_r_*.

The development of ultimate load (*P_u_*) with varying *ρ_r_* is presented in Figure 17b. The ultimate load depends on the strength and stiffness of tensile rebars. EPC beams with CFRP rebars result in greater ultimate load than those with steel rebars, especially at low *ρ_r_* levels, because of the higher ultimate tensile rebar stresses developed in CFRP rebars. Meanwhile, EPC beams with GFRP rebars exhibit a larger ultimate load at low *ρ_r_* levels (e.g., *ρ_r_* = 0.5%) but a smaller one at high *ρ_r_* levels (e.g., *ρ_r_* = 1.98%), when compared to those with steel rebars.

The development of ultimate tendon stress increment (Δ*σ_p_*) against *ρ_r_* is illustrated in Figure 17c. In EPC beams with GFRP rebars, Δ*σ_p_* decreases slowly in a linear manner as *ρ_r_* rises, whereas, in EPC beams with CFRP rebars, it decreases substantially. The reductions in Δ*σ_p_* in EPC beams containing CFRP and GFRP rebars, when *ρ_r_* increases from 0.5 to 1.98%, are 288.97 and 214.09 MPa, respectively. FRP rebars (especially GFRP) result in higher Δ*σ_p_* than steel rebars.

**Figure 18:**
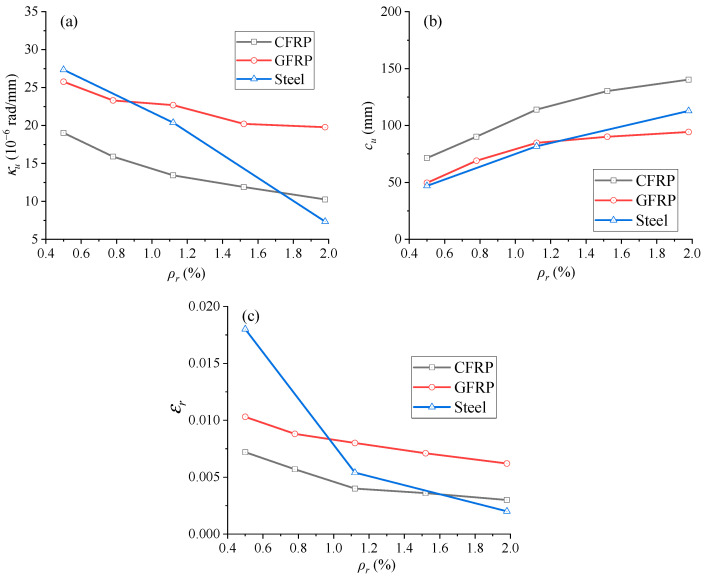
Ultimate behavior with varying reinforcement ratio. (**a**) ultimate curvature; (**b**) ultimate neutral axis depth; (**c**) ultimate tensile rebar strain.

Figure 18a shows the development of ultimate midspan curvature (*κ_u_*) with different *ρ_r_* levels. From the figure, it is evident that *κ_u_* decreases as *ρ_r_* rises. When *ρ_r_* is less than 0.9%, EPC beams with GFRP rebars have less *κ_u_* than those with steel ones. Observations are opposite when *ρ_r_* is greater than 0.88%. CFRP rebars generally cause lower *κ_u_* than steel ones, except when *ρ_r_* > 1.73%.

Variations of ultimate neutral axis depth (*c_u_*) and ultimate tensile rebar strain (*ε_r_*) against *ρ_r_* are shown in Figure 18b,c, respectively. The *c_u_* and *ε_r_* exhibit opposite trends with varying *ρ_r_*, i.e., increasing *ρ_r_* causes an increment for *c_u_* but a reduction for *ε_r_*. Additionally, EPC beams with CFRP rebars have higher *c_u_* than those with GFRP or steel rebars. This suggests that CFRP rebars may cause lower ductile ability of the structure compared to GFRP or steel rebars. With increasing *ρ_r_*, the *c_u_* difference between EPC beams with CFRP and GFRP rebars increases progressively, from 21.79 mm to 45.97 mm. Due to the non-existence of yield point in FRP bars, the *ε_r_* in CFRP or GFRP rebars exhibits a consistently decreasing trend with increasing *ρ_r_*.

## 4. Assessment of Typical Models for Predicting the Ultimate Tendon Stress

### 4.1. Typical Models

In EPC beams, the external tendons are not structurally bonded to the main concrete element but are connected solely through anchorages and deviators. The lack of direct attachment prevents coordinated deformation between the tendons and the concrete, leading to a uniform stress distribution along the tendon.

Externally prestressed flexural members, provided second-order effects are minimized, exhibit similar behavior to that of internally unbonded prestressed flexural members. The ultimate tendon stress is an essential factor in evaluating the flexural strength of EPC members. Because of similar stress increment behavior of EPC members and unbonded prestressed concrete members, global design standards for external prestressing largely follow unbonded prestressing principles, with adjustments through construction techniques and tendon stress corrections.

In designing EPC beams, a key parameter often employed to predict Δ*σ_p_* is the combined reinforcement index, which reflects the relative height of the compression zone and the rotational capacity of the cross-section, making it the most influential factor in determining the tendon stress increment. As the tensile stress in FRP rebars is generally far below rupture strengths, this index (for EPC T-beams with FRP rebars) is expressed as follows [43]:

(5) $$\omega_{0}=\frac{A_{p}\sigma_{pe}+A_{r}\sigma_{r}-f_{c,r}(b_{f}^{\prime }-b)h_{f}^{\prime }}{f_{c,r}bd_{p}}$$

where *A_p_* and *A_r_* are the areas of tendons and tensile rebars, respectively; *σ_pe_* and *σ_r_* are the effective prestress of tendons and the ultimate stress of tensile rebars, respectively; $b_{f}^{\prime }$ and *b* are the flange width and web thickness, respectively; $h_{f}^{\prime }$ and *d_p_* are the flange thickness and the tendon depth, respectively; *f_c_*_,*r*_ is concrete cylinder compressive strength.

JGJ/T 92-93 [44] proposed the following equation to predict Δ*σ_p_*:

(6a) $$\Delta \sigma_{p}=500-770\omega_{0}$$

for *L*/*d_p_* ≤ 35, *ω*_0_ ≤ 0.45; and

(6b) $$\Delta \sigma_{p}=250-380\omega_{0}$$

for *L*/*d_p_* > 35, *ω*_0_ ≤ 0.45.

JGJ 92-2016 [43] introduced a modified version of the previous equation, expressed as follows:

(7) $$\Delta \sigma_{p}=(240-335\omega_{0})(0.45+5.5h/L_{0})\frac{L_{l}}{L_{t}}$$

where *L*_0_ is the calculated span length; *L_t_* is the tendon length between anchorages; and *L_l_* is the length of loaded spans; *ω*_0_ is not greater than 0.4; *h* is the cross-sectional depth.

By using a numerical program neglecting the bond–slip effect of FRP rebars, Pang et al. [23] carried out a parametric assessment of mechanical and deformation characteristics of simple EPC beams with FRP rebars and introduced the following equation for predicting Δ*σ_p_* in these beams:

(8) $$\Delta \sigma_{p}=626-1032\omega_{0}$$

Apart from the index *ω*_0_, the neutral axis depth is another parameter widely employed by design codes. To predict Δ*σ_p_*, AASHTO-1994 [45] employed the following equation:

(9) $$\Delta \sigma_{p}=\Omega_{u}E_{p}\varepsilon_{c,u}\left(\frac{d_{p}}{c_{u}}-1\right)\frac{L_{l}}{L}$$

where *E_p_* and *d_p_* are the elastic modulus and depth of tendons, respectively; *ε_c_*_,*u*_ is the ultimate concrete compressive strain; *c_u_* is the neutral axis depth at failure; *L* and *L_l_* are the total span length and length of loaded spans, respectively. Equation (10) is formulated using the strain compatibility method, similar to that for bonded tendons, and incorporating Ω*_u_* to account for the effects of unbonded tendons.

(10) $$\Omega_{u}=\frac{{\left(\Delta \varepsilon_{ub}\right)}_{avg}}{{\left(\Delta \varepsilon_{b}\right)}_{\max}}$$

where Ω*_u_* is the bond reduction coefficient; (Δ*ε_ub_*)*_avg_* and (Δ*ε_b_*)*_max_* are the average variations of concrete strain in unbonded prestressed concrete members and the variation of concrete strain on the critical section in equivalent bonded prestressed concrete members, respectively.

Furthermore, AASHTO-2017 [46] suggested a deformation-based model, initially proposed in [47], which is expressed by

(11) $$\Delta \sigma_{p}=6200\left(\frac{d_{p}-c_{u}}{l_{e}}\right)$$

where

(12) $$l_{e}=\frac{2L_{t}}{2+N_{s}}$$

where *l_e_* and *l_t_* are the effective length of external unbonded tendons and the tendon length between anchorages, respectively; *N_s_* is the number of plastic hinges in the structure at the ultimate state. It is noteworthy that plastic hinges do not form in EPC beams with FRP rebars, which could limit the suitability of Equation (11) for these beams.

### 4.2. Comparative Analysis

**Figure 19:**
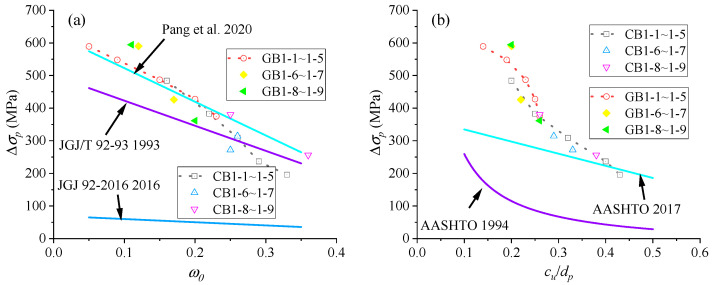
Numerical and code predictions on Δ*σ_p_*. (**a**) variation in Δ*σ_p_* based on *ω*_0_; (**b**) variation in Δ*σ_p_* based on *c_u_*/*d_p_* [23,43,44,45,46].

Figure 19a shows the numerically obtained Δ*σ_p_*−*ω*_0_ development for EPC beams with FRP rebars, as well as relationship curves by JGJ/T 92-93 [44], JGJ/T 92-2016 [43], and the Pang et al. model [23]. FEM data, along with AASHTO-1994 [45] and 2017 [46] concerning the Δ*σ_p_*−*c_u_*/*d_p_* development, are shown in Figure 19b. A comparison of FEM data with predictions by different simplified models is included in Table 7.

From Figure 19a, it is seen that, based on numerical findings, Δ*σ_p_* in EPC beams with FRP rebars decreases with an increase in *ω*_0_, which ranges from 0.16 to 0.36 when CFRP rebars are used and from 0.05 to 0.23 when GFRP rebars are used. The prediction of Δ*σ_p_* by JGJ 92-2016 is significantly lower than FEM data, indicating this code is too conservative. JGJ/T 92-93 demonstrates reasonable accuracy in predicting Δ*σ_p_* in EPC beams with FRP rebars, particularly when *ω*_0_ exceeds 0.2. This code generally exhibits a degree of conservatism. The Pang et al. model [23] shows a good agreement with FEM data in predicting the Δ*σ_p_* of the EPC beams with FRP rebars, especially at low levels of *ω*_0_. The observed discrepancy may be attributed to the different bond–slip relationships between FRP rebars and concrete.

As demonstrated in Figure 19b and Table 7, the *c_u_*/*d_p_* of EPC beams with CFRP rebars ranges from 0.22 to 0.43 for EPC beams with CFRP rebars, while it ranges from 0.14 to 0.26 for those with GFRP rebars. The findings indicate that the AASHTO-1994 provides poor forecasts, characterized by a tendency toward excessive conservatism. AASHTO-2017 is superior to AASHTO-1994 in terms of the predictive results, but it is overly conservative at low values of *c_u_*/*d_p_*.

**Figure 20:**
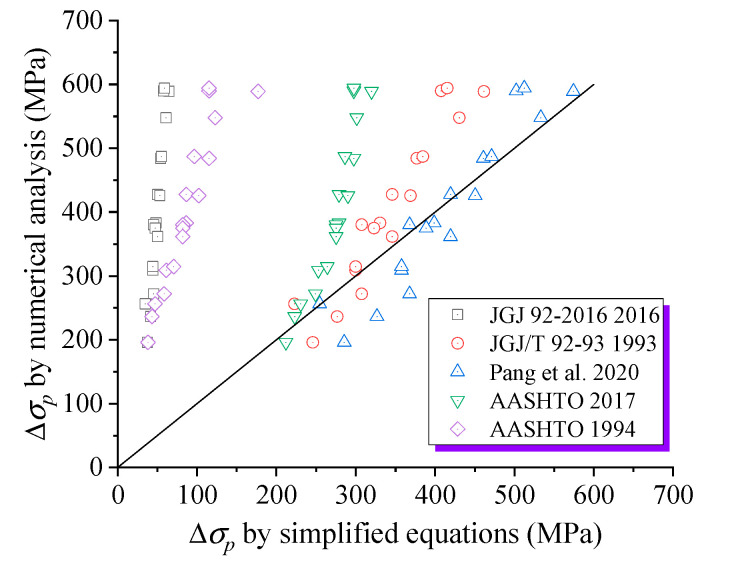
Comparison of Δ*σ_p_* by simplified models with numerical predictions [23,43,44,45,46].

Figure 20 and Table 7 compare various simplified models for predicting Δ*σ_p_* with FEM, revealing that JGJ 92-2016 and AASHTO-1994 produce the least accurate predictions, often overly conservative. AASHTO-2017 shows a significant deviation, indicating a relatively poor correlation with the numerical results. JGJ/T 92-93 exhibits a certain deviation in calculating the higher tendon stress increment, while the overall consistency remains satisfactory. The Pang et al. [23] model offers better accuracy in predicting the Δ*σ_p_* for EPC beams with FRP rebars, compared to the code models previously mentioned, despite its omission of the bond–slip interaction between FRP rebars and concrete.

### 4.3. Applicability to Real-World Engineering Structures

The findings of this study, which focus on the bond–slip interactions between FRP rebars and concrete in simple CFRP tendon–EPC beams, provide valuable insights for the design and analyses of real-world engineering structures such as bridges and buildings. They underscore the significant impacts of bond–slip behavior on the mechanical performance of EPC beams with FRP rebars, emphasizing the importance of incorporating this effect into structural design to achieve more accurate predictions of tendon stress and load-carrying capacity.

In practical engineering applications, especially in bridge construction and tower block retrofitting, FRP materials are increasingly favored due to their high strength, corrosion resistance, and lightweight properties. However, the bond–slip behavior between FRP rebars and concrete can profoundly influence structural performance, particularly under dynamic and long-term loading conditions. The stress degradation observed in FRP rebars as a result of bond–slip effects, as highlighted in this study, is crucial for ensuring the long-term durability and safety of structures reinforced with FRP materials.

When applying the findings of this study to practical engineering scenarios, it is essential to recognize the limitations of current design models. The study compares several existing models, such as JGJ 92-2016 [43], JGJ/T 92-93 [44], AASHTO-1994 [45], AASHTO-2017 [46], and the Pang et al. [23] model, for predicting the ultimate tendon stress increase (Δ*σ_p_*) in EPC beams with FRP rebars. The results reveal the following:

JGJ 92-2016 [43] and AASHTO-1994 [45] provide the least accurate predictions, with most of their results being overly conservative. These models tend to underestimate the actual stress increase, which may lead to overly cautious designs that fail to fully leverage the potential of FRP materials.

JGJ/T 92-93 [44] demonstrates reasonable accuracy of tendon stress predictions, particularly when the combined reinforcement index (denoted by ω₀) exceeds 0.2. This model strikes a better balance between safety and material efficiency, providing more reliable predictions for EPC beams with FRP rebars, though it still neglects the consideration of bond–slip effects.

AASHTO-2017 [46] exhibits a significant deviation from the numerical results, indicating a relatively poor correlation with the actual behavior of EPC beams with FRP rebars. This deviation highlights the need to improve design models to account for more complex material interactions, such as bond–slip.

The Pang et al. model [23] offers the highest accuracy in predicting Δ*σ_p_* for EPC beams with FRP rebars, although it does not account for the bond–slip interaction between FRP rebars and concrete. Despite this limitation, the model outperforms others in terms of accuracy, underscoring the importance of refining existing design codes to incorporate bond–slip effects for more reliable structural predictions.

This study reveals that current design models often fail to fully consider bond–slip behavior, leading to less accurate tendon stress predictions in EPC beams. Although the Pang et al. model is the most accurate among those evaluated, future design codes must integrate bond–slip effects to enhance prediction reliability. The refined 3D FEM introduced in this study provides engineers with a robust tool for predicting the mechanical behavior of EPC beams with FRP rebars. By incorporating bond–slip effects, the model offers a more precise simulation of the FRP rebars–concrete interface, facilitating improved design decisions, particularly for structures exposed to cyclic loading, temperature variations, and other environmental challenges.

In bridge construction and high-rise buildings, safety and cost efficiency are paramount. Incorporating bond–slip effects into design models can significantly enhance the performance and lifespan of FRP-reinforced structures, reducing the risk of underestimating material stresses and improving structural reliability. Furthermore, this study highlights the critical need to refine existing design codes to address bond–slip interactions between FRP rebars and concrete, particularly in the context of EPC beams. The findings contribute valuable insights to the optimal design of EPC beams with FRP reinforcement, laying the groundwork for engineering solutions that are safer, more durable, and cost-effective.

## 5. Conclusions

The study yields the following conclusions:

- A high-fidelity 3D FEM for EPC beams with FRP rebars is developed. The interaction between FRP rebars and concrete is considered by implementing a self-designed program that links FRP rebar nodes to concrete and incorporates appropriate bond–slip relationships. The model effectively captures the slip behavior of the FRP rebars. Its reliability is validated by comparing simulation outcomes with experimental data.
- There is a specific phenomenon of stress degradation of FRP rebars due to the bond–slip effect. This effect reduces the structural stiffness and load-carrying capacity and may lead to interface failure due to stress concentration. Thus, the bond–slip effect is essential for the accurate simulation and design of EPC beams with FRP rebars.
- As *ρ_r_* in EPC beams with CFRP rebars increases, Δ*_u_*, Δ*σ_p_*, *ε_r_*, and *κ_u_* tend to decrease, while *P_u_* and *c_u_* tend to increase. Within *ρ_r_* studied, EPC beams with CFRP rebars exhibit greater *P_u_*, Δ*_u_*, *c_u_*, and Δ*σ_p_* compared to EPC beams with steel rebars. The difference in *c_u_* between EPC beams with CFRP and GFRP rebars gradually increases with increasing *ρ_r_*. Additionally, enhancing concrete grade significantly improves structural stiffness and ultimate bearing capacity.
- JGJ 92-2016 and AASHTO-1994 yield the poorest results, being significantly over-conservative, while JGJ/T 92-93 demonstrates reasonable accuracy in predicting Δ*σ_p_* in EPC beams with FRP rebars, particularly when *ω*_0_ exceeds 0.2. AASHTO-2017 shows a relatively poor correlation with the numerical results. The Pang et al. model offers better accuracy in predicting the Δ*σ_p_* for EPC beams with FRP rebars than the aforementioned design codes, although it ignores the bond–slip interaction between FRP rebars and concrete.

## Figures and Tables

**Table 1:** Material parameters for specimens.

Beam	Compressive Steel			Stirrup			Concrete
	*D*_1_ (mm)	*E_s_*_1_ (GPa)	*f_y_*_1_ (MPa)	*D*_2_ (mm)	*E_s_*_2_ (GPa)	*f_y_*_2_ (MPa)	*f_cu_* (MPa)
BL1-2 (P-GFRP)	14	200	380	8	210	308	31.5
TL1-2 (R-CFRP)	16	200	380	12	210	308	31.5
BL2-1 (R-GFRP)	12	200	380	10	210	308	31.5

Note: P-GFRP, R-GFRP, and R-CFRP = plain GFRP, ribbed GFRP, and ribbed CFRP, respectively; *D*_1_, *E_s_*_1_, and *f_y_*_1_ = diameter, Young’s modulus, and yield strength of compressive steel, respectively; *D*_2_, *E_s_*_2_, and *f_y_*_2_ = diameter, Young’s modulus, and yield strength of stirrup, respectively; *f_c__u_* = cubic compressive strength (measured value).

**Table 2:** Comparison of the experimental results and computational evaluation.

Beam	Test [38]		Analysis Considering Bond–Slip				Analysis Neglecting Bond–Slip			
	UL (kN)	UD (mm)	UL (kN)	Error (%)	UD (mm)	Error (%)	UL (kN)	Error (%)	UD (mm)	Error (%)
TL1-2	239.22	40.17	226.50	−5.32	40.33	0.40	281.73	17.77	41.69	3.78
BL1-2	96.39	47.78	104.50	8.41	47.43	−0.73	-	-	-	-
BL2-1	88.66	20.94	93.31	5.24	22.61	7.98	112.27	23.61	23.78	13.56

Note: UL and UD = ultimate load and deflection, respectively.

**Table 3:** Material properties of specimens.

Beam	Steel Rebars						External Tendons				Concrete
	*A_s_* (mm^2^)	*f_y_* (MPa)	*E_s_* (GPa)	$A_{s}^{,}$ (mm^2^)	$f_{y}^{,}$ (MPa)	$E_{s}^{,}$ (GPa)	*A_p_* (mm^2^)	*E_p_* (GPa)	*f_pu_* (MPa)	*σ_pe_* (MPa)	*f_c_*_,*r*_ (MPa)
T-0	402	530	210	201	338	180	110.2	193	1900	1297	34.6
T-1	402	530	210	201	338	180	110.2	193	1900	1197	34.2
T-2	402	530	210	201	338	180	110.2	193	1900	1182	28.7

Note: *A_s_*, *f_y_*, and *E_s_* = area, yield strength, and Young’s modulus of deformed steel rebars, respectively; $A_{s}^{,}$, $f_{y,}^{,}$ and $E_{s}^{,}$ = area, yield strength, and Young’s modulus of mild steel rebars, respectively; *A_p_* = tendon area; *E_p_* and *f_pu_* = Young’s modulus and tensile strength of external tendons, respectively; *σ_pe_* = effective prestress; *f_c_*_,*r*_ = concrete cylinder compressive strength.

**Table 4:** Comparison of the test results and numerical evaluation.

Beam	Ultimate Deflection (mm)			Ultimate Tendon Stress (MPa)		
	Test	Numerical	Error (%)	Test	Numerical	Error (%)
T-0	34.62	35.69	3.09	1668.32	1628.34	−2.40
T-1	31.32	32.87	4.95	1648.43	1600.18	−2.93
T-2	40.51	41.73	3.01	1665.62	1611.17	−3.27

**Table 5:** Material parameters of the research model.

Contents	Beam	Tensile Rebars				Compressive Rebars				Stirrup (CFRP)		Tendons (CFRP)			Concrete
		Type	*E_t_* (GPa)	*D_t_* (mm)	*ρ_r_* (%)	Type	*E_n_* (GPa)	*D_c_* (mm)	*ρ_c,r_* (%)	*E_s_* (GPa)	*D_s_* (mm)	*E_p_* (GPa)	*D_p_* (mm)	*σ_pe_* (MPa)	*f_cu_* (MPa)
Group A	CB1-1	CFRP	145	16	0.50	CFRP	145	10	0.33	147	8	160	12	1060	40
	CB1-2	CFRP	145	20	0.78	CFRP	145	10	0.33	147	8	160	12	1060	40
	CB1-3	CFRP	145	24	1.12	CFRP	145	10	0.33	147	8	160	12	1060	40
	CB1-4	CFRP	145	28	1.52	CFRP	145	10	0.33	147	8	160	12	1060	40
	CB1-5	CFRP	145	32	1.98	CFRP	145	10	0.33	147	8	160	12	1060	40
	CB1-6	CFRP	130	24	1.12	CFRP	145	10	0.33	147	8	160	12	1060	40
	CB1-7	CFRP	160	24	1.12	CFRP	145	10	0.33	147	8	160	12	1060	40
	CB1-8	CFRP	145	24	1.12	CFRP	145	10	0.33	147	8	160	12	1060	30
	CB1-9	CFRP	145	24	1.12	CFRP	145	10	0.33	147	8	160	12	1060	50
Group B	GB1-1	GFRP	55	16	0.50	GFRP	55	10	0.33	147	8	160	12	1060	40
	GB1-2	GFRP	55	20	0.78	GFRP	55	10	0.33	147	8	160	12	1060	40
	GB1-3	GFRP	55	24	1.12	GFRP	55	10	0.33	147	8	160	12	1060	40
	GB1-4	GFRP	55	28	1.52	GFRP	55	10	0.33	147	8	160	12	1060	40
	GB1-5	GFRP	55	32	1.98	GFRP	55	10	0.33	147	8	160	12	1060	40
	GB1-6	GFRP	40	24	1.12	GFRP	55	10	0.33	147	8	160	12	1060	40
	GB1-7	GFRP	70	24	1.12	GFRP	55	10	0.33	147	8	160	12	1060	40
	GB1-8	GFRP	55	24	1.12	GFRP	55	10	0.33	147	8	160	12	1060	30
	GB1-9	GFRP	55	24	1.12	GFRP	55	10	0.33	147	8	160	12	1060	50
Group C	SB1-1	Steel	200	16	0.50	Steel	200	10	0.33	147	8	160	12	1060	40
	SB1-2	Steel	200	24	1.12	Steel	200	10	0.33	147	8	160	12	1060	40
	SB1-3	Steel	200	32	1.98	Steel	200	10	0.33	147	8	160	12	1060	40

Note: *E_t_*, *E_n_, E_s_*, and *E_p_* = elastic modulus of tensile rebars, compressive rebars, stirrup and tendons, respectively; *D_t_*, *D_c_, D_s_*, and *D_p_* = diameters of tensile rebars, compressive rebars, stirrup and tendons, respectively; *ρ_r_* and *ρ_c,r_* = ratio of tensile and compressive rebars, respectively; *σ_pe_* = effective prestress; *f_cu_* = cubic compressive strength of concrete.

**Table 6:** Ultimate behavior of EPC beams.

Group	Beam	Ultimate Behavior					
		Deflection (mm)	Load (kN)	Tendon Stress Increase (MPa)	Curvature (10^−6^ rad/mm)	Neutral Axis Depth (mm)	Tensile Rebar Strain
A	CB1-1	115.99	274.57	484.19	19.03	71.41	0.0072
	CB1-2	101.42	297.12	382.93	15.91	90.22	0.0057
	CB1-3	90.91	313.06	308.80	13.44	114.01	0.0040
	CB1-4	85.07	324.03	236.42	11.90	130.32	0.0036
	CB1-5	73.41	336.92	195.93	10.26	140.27	0.0030
	CB1-6	100.94	311.26	314.54	14.4	102.83	0.0051
	CB1-7	73.76	301.64	271.91	12.38	118.70	0.0036
	CB1-8	75.47	274.77	256.28	9.92	137.71	0.0034
	CB1-9	120.92	367.70	380.24	15.88	93.61	0.0045
B	GB1-1	120.95	206.51	588.98	25.76	49.62	0.0103
	GB1-2	119.69	229.49	547.89	23.30	69.10	0.0088
	GB1-3	120.01	252.65	486.93	22.69	84.79	0.0080
	GB1-4	116.59	273.08	427.62	20.21	90.16	0.0071
	GB1-5	114.59	285.76	374.89	19.78	94.30	0.0062
	GB1-6	140.78	240.32	589.85	17.57	72.89	0.0098
	GB1-7	107.71	261.78	425.86	15.51	80.46	0.0069
	GB1-8	84.96	206.29	361.53	17.41	94.02	0.0060
	GB1-9	135.86	282.96	594.26	24.12	72.59	0.0091
C	SB1-1	79.97	166.62	166.74	27.35	46.95	0.0180
	SB1-2	69.08	248.21	194.85	20.37	81.79	0.0054
	SB1-3	69.67	323.04	117.80	7.34	112.84	0.0020

**Table 7:** Comparison of the Δ*σ_p_* values predicted by numerical analysis vs. those from simplified equations.

RaberType	Beam	*ρ_r_* (%)	*ω*_0_	*c_u_*/*d_p_*	Δ*σ_p_* (MPa)						(Δ*σ_p_*)_Equation_/(Δ*σ_p_*)_Numerical_				
					Num	JGJ16	JGJ92	Pang	AO17	AO94	JGJ16	JGJ92	Pang	AO17	AO94
CFRP	CB1-1	0.5	0.16	0.22	484.19	54.06	376.80	460.88	297.60	115.20	0.11	0.78	0.95	0.61	0.24
	CB1-2	0.78	0.22	0.25	382.93	48.23	330.60	398.96	279.00	86.40	0.13	0.86	1.04	0.73	0.23
	CB1-3	1.12	0.26	0.32	308.80	44.34	299.80	357.68	252.96	61.20	0.14	0.97	1.16	0.82	0.20
	CB1-4	1.52	0.29	0.4	236.42	41.43	276.70	326.72	223.20	43.20	0.18	1.17	1.38	0.94	0.18
	CB1-5	1.98	0.33	0.43	195.93	37.54	245.90	285.44	212.04	38.17	0.19	1.26	1.46	1.08	0.19
	CB1-6	1.12	0.26	0.29	314.54	44.34	299.80	357.68	264.12	70.51	0.14	0.95	1.14	0.84	0.22
	CB1-7	1.12	0.25	0.33	271.91	45.31	307.50	368.00	249.24	58.47	0.17	1.13	1.35	0.92	0.22
	CB1-8	1.12	0.36	0.38	256.28	34.63	222.80	254.48	230.64	46.99	0.14	0.87	0.99	0.90	0.18
	CB1-9	1.12	0.25	0.26	380.24	45.31	307.50	368.00	275.28	81.97	0.12	0.81	0.97	0.72	0.22
GFRP	GB1-1	0.5	0.05	0.14	588.98	64.74	461.50	574.40	319.92	176.91	0.11	0.78	0.98	0.54	0.30
	GB1-2	0.78	0.09	0.19	547.89	60.85	430.70	533.12	301.32	122.78	0.11	0.79	0.97	0.55	0.22
	GB1-3	1.12	0.15	0.23	486.93	55.03	384.50	471.20	286.44	96.42	0.11	0.79	0.97	0.59	0.20
	GB1-4	1.52	0.2	0.25	427.62	50.17	346.00	419.60	279.00	86.40	0.12	0.81	0.98	0.65	0.20
	GB1-5	1.98	0.23	0.26	374.89	47.26	322.90	388.64	275.28	81.97	0.13	0.86	1.04	0.73	0.22
	GB1-6	1.12	0.12	0.20	589.85	57.94	407.60	502.16	297.60	115.20	0.10	0.69	0.85	0.50	0.20
	GB1-7	1.12	0.17	0.22	425.86	53.08	369.10	450.56	290.16	102.11	0.12	0.87	1.06	0.68	0.24
	GB1-8	1.12	0.20	0.26	361.53	50.17	346.00	419.60	275.28	81.97	0.14	0.96	1.16	0.76	0.23
	GB1-9	1.12	0.11	0.20	594.26	58.91	415.30	512.48	297.60	115.20	0.10	0.70	0.86	0.50	0.19

Note: Num = Numerical results; JGJ16 = JGJ 92-2016 [43]; JGJ92 = JGJ/T 92-93 [44]; Pang = Pang et al. model [23]; AO17 = AASHTO-2017 [46]; AO94 = AASHTO-1994 [45].

## Data Availability

The original contributions presented in this study are included in the article. Further inquiries can be directed to the corresponding author.
